# Antioxidant Capacity of Colostrum of Mothers with Gestational Diabetes Mellitus—A Cross-Sectional Study

**DOI:** 10.3390/nu17213324

**Published:** 2025-10-22

**Authors:** Paulina Gaweł, Karolina Karcz, Natalia Zaręba-Wdowiak, Barbara Królak-Olejnik

**Affiliations:** 1 Department of Neonatology, Wroclaw Medical University, 50-556 Wroclaw, Poland; k.karcz@umw.edu.pl (K.K.); barbara.krolak-olejnik@umw.edu.pl (B.K.-O.); 2 Department and Institute of Pharmaceutical Biochemistry, Wroclaw Medical University, Borowska 211 A, 50-556 Wroclaw, Poland; natalia.zareba-wdowiak@umw.edu.pl

**Keywords:** gestational diabetes, colostrum, antioxidant capacity

## Abstract

**Background:** Women with gestational diabetes mellitus (GDM) are vulnerable to oxidative stress, yet limited data exist on the antioxidant potential of their breast milk. This study aimed to evaluate the antioxidant capacity and basic composition of colostrum in women with GDM compared to healthy controls, focusing on total antioxidant capacity (TAC) and enzymatic antioxidants: superoxide dismutase (SOD), catalase (CAT), and glutathione peroxidase (GPx). **Methods:** The study included 77 lactating mothers: 56 with gestational diabetes (15 managed with diet/exercise—GDM G1; 41 required insulin—GDM G2) and 21 healthy controls. Colostrum samples were collected on days 3–5 postpartum and analyzed for macronutrients and antioxidant enzymes. To enable comparisons across study groups and to explore associations with maternal characteristics, a range of statistical methods was applied. A taxonomic (classification) analysis was then performed using the predictors that best fit the data: study group membership, maternal hypothyroidism history (from the medical interview), and gestational weight gain. **Results:** TAC was significantly lower in the GDM G2 group compared to GDM G1 and controls (*p* = 0.001), with no differences in enzymatic antioxidants. The control group had the highest energy (*p* = 0.048) and dry matter content (*p* = 0.015), while protein, fat, and carbohydrate levels did not differ significantly. After dividing the study group into four clusters, based on maternal health factors, including GDM status and thyroid function, TAC levels differed significantly between clusters, with the highest values observed in Cluster 3 (healthy controls without thyroid dysfunction) and the lowest in Cluster 2 (GDM and hypothyroidism). Analysis of colostrum composition revealed significant differences in energy content (*p* = 0.047) and dry matter concentration (*p* = 0.011), while no significant differences were found in other macronutrients. **Conclusions:** Our findings suggest that maternal metabolic and endocrine conditions, such as GDM and thyroid dysfunction, may differentially influence the nutritional and functional properties of colostrum—particularly its antioxidant potential.

## 1. Introduction

Gestational Diabetes Mellitus (GDM) is a complex and multifactorial metabolic disorder defined as a “diabetes first diagnosed in the second or third trimester of pregnancy that is not clearly overt diabetes prior to gestation” [1]. GDM is estimated to affect approximately 14% of pregnancies worldwide, and its prevalence continues to rise in parallel with the global obesity epidemic. The rate of GDM varies significantly depending on the population’s risk factors as well as differences in screening methods and diagnostic criteria [2]. The condition is associated with various adverse maternal, fetal, and perinatal outcomes. Women affected by GDM experience higher rates of adverse perinatal outcomes, including caesarean section, shoulder dystocia, and are more likely to develop hypertension and preeclampsia during pregnancy [3]. Elevated levels of blood glucose have indeed been shown to have a detrimental effect on the immune system, resulting in an increased susceptibility to various infections, including urinary tract infections (UTIs) and endometritis. Moreover, there is evidence to suggest that this condition may potentially increase the risk of significant bleeding and perineal tears during or after childbirth [4]. In the long term, there is an increased risk of developing type-2 diabetes mellitus, cardiovascular disease (CVD) and a high risk of GDM recurring with further pregnancies [5]. For the offspring, maternal diabetes is linked to increased birth weight, with a higher likelihood of large-for-gestational-age (LGA) infants and macrosomia. Also they are at greater risk for delivery complications, including neonatal hypoglycemia, polihydramnios, stillbirth, perinatal injuries. Excess insulin has been demonstrated to delay surfactant production, thus elevating the risk of respiratory distress syndrome (RDS). It has been hypothesized that placental dysfunction may be a contributing factor to fetal hypoxia during labor. As evidenced by research, GDM has been shown to slightly raise the risk of cardiovascular, neurological, and other developmental defects. While the magnitude of this increase is less significant when compared with that associated with pre-existing diabetes, further investigation is warranted to fully elucidate the underlying mechanisms and potential long-term consequences [6,7]. From a long-term perspective, infants exposed to GDM have an increased risk of obesity and insulin resistance during childhood, along with an elevated incidence of impaired glucose tolerance and type 2 diabetes in adulthood [8,9,10].

Recent studies have shown that various maternal metabolic and endocrine disorders can significantly influence the composition of human milk. For instance, GDM has been associated with substantial alterations in the lipid profile of breast milk, including increases in phospholipids and sphingolipids and decreases in neutral lipids, many of which correlate with maternal glucose levels and infant growth parameters [11].

Beyond diabetes, recent evidence also suggests that thyroid dysfunction—particularly gestational or chronic hypothyroidism—can influence the nutritional and proteomic composition of human milk. Proteomic analyses of colostrum from mothers with gestational hypothyroidism have revealed downregulation of several metabolic and cytoskeletal proteins (e.g., GAPDH, actin, enzymes of glycolysis and gluconeogenesis), alongside upregulation of certain immune-related proteins. Moreover, milk from hypothyroid mothers has been found to contain lower protein and fat levels compared with milk from mothers with normal thyroid function during pregnancy [12]. Additionally, maternal weight factors—such as pre-pregnancy BMI, obesity, and excessive gestational weight gain—are associated with variations in breast milk macronutrient and energy content, potentially reflecting the broader metabolic status of the mother [13].

Recent studies also highlight that breastfeeding offers significant health benefits for both mother and infant, with particular importance for women with gestational diabetes [14]. Breastfeeding is the recommended mode of feeding, providing nutritional, immunological, and developmental benefits. The World Health Organization (WHO), American Academy of Pediatrics (AAP), European Society for Paediatric Gastroenterology, Hepatology and Nutrition (ESPGHAN) recommends exclusively breastfeeding for the first six months of a baby’s life continuing for up to two years and beyond, with the introduction of complementary foods at six months [15,16,17].

For infants born to mothers with GDM, human milk presents an important factor to mitigate the potential consequences of GDM—likely through compositional changes [18]. Moreover, human milk exhibits potent antioxidant activity, which is particularly relevant in the context of GDM where both mothers and their babies are exposed to significantly elevated oxidative stress [19]. Although pregnancy itself is considered a ‘diabetogenic state’ characterized by increased metabolic activity and subsequent oxidative stress, GDM further intensifies this process. Oxidative stress results from an imbalance between the production of reactive oxygen species (ROS) and the antioxidant defense mechanisms of the human body. ROS are oxygen-containing molecules with one or more unpaired electrons or those readily oxidized into free radicals, which can cause molecular and tissue damage and have been linked to various chronic diseases [20].

Human milk, among its many bioactive components, serves as a significant source of antioxidants, which play a crucial role in maintaining redox homeostasis and protecting the infant from oxidative stress, particularly during the early postnatal period when endogenous antioxidant systems are still immature. Antioxidants in human milk have been identified as probable systemic defense mechanisms against oxidative stress in infants, particularly within the gastrointestinal tract [21,22]. Human milk contains a wide array of both fat- and water-soluble vitamins at physiologically relevant concentrations. Several of these vitamins—such as vitamins A, C, and E, as well as carotenoids—are well-established antioxidants with the capacity to neutralize reactive oxygen species (ROS) and limit oxidative damage. Beyond these micronutrients, human milk is also enriched with numerous non-nutrient bioactive compounds exhibiting antioxidant activity. Among these, enzymatic antioxidants such as superoxide dismutase (SOD), catalase (CAT), and glutathione peroxidase (GPx) are of particular interest [23]. These enzymes form an integral part of the milk’s antioxidant defense system and can be quantitatively assessed to evaluate the overall antioxidative potential of human milk.

Many comparative studies agree that no single test can comprehensively assess antioxidant capacity; hence, it is common practice to apply multiple complementary assays [24]. Total Antioxidant Capacity (TAC) refers to the overall ability of a biological sample to neutralize ROS and other reactive species. TAC reflects the combined effect of both enzymatic and non-enzymatic antioxidants, while measurements of specific antioxidant enzymes such as SOD, CAT, GPx provide more detailed insight into antioxidant defense mechanisms [24,25].

Although women with GDM are known to be vulnerable to oxidative stress, there is limited literature evaluating this condition through the analysis of the antioxidant potential of human milk as a nutritional source for the newborn/infant, including its potential protective role against oxidative stress in infants. Therefore, the aim of this study was to assess the antioxidant capacity of human milk, precisely colostrum, in mothers with GDM compared to normoglycemic controls. In addition, specific high-molecular-weight antioxidants—including superoxide dismutase, catalase, and glutathione peroxidase—were quantified to provide a more comprehensive understanding of the milk’s antioxidative profile.

## 2. Materials and Methods

### 2.1. Study Design

Our cross-sectional observational study involved lactating mothers with gestational diabetes mellitus (GDM) and normoglycemic healthy control. Participants were enrolled from June 2024 to November 2024. The study was approved by the Bioethics Committee of the Medical University of Wroclaw (agreement No. 378/2023N) according to Helsinki declarations involving human subjects. All mothers gave written informed consent before study procedures were performed.

### 2.2. Study Group

A total of 77 women were involved in the study, including 56 mothers with GDM. 17 mothers in GDM were treated with diet and physical activity (GDM G1), whereas 39 women were administered insulin therapy. The remaining 21 were enrolled as a control group with no history of glucose intolerance before or during pregnancy. The mothers were patients at the 2nd Department of Gynecology and Obstetrics and their babies were hospitalized after birth in the Department of Neonatology at the University Teaching Hospital, Wrocław, Poland. The inclusion criteria were as follows: (1) maternal age 18–45 years, (2) delivery at ≥35 + 0/7 weeks of gestation, (3) vaginal delivery or cesarean section, (4) singleton pregnancy, (5) good postnatal condition of the newborn measured by Apgar score > 7 points, (6) exclusive or predominant breastfeeding, (7) mothers’ informed consent, (8) recruitment within first 5 days after birth. The exclusion criteria were: (1) any maternal or neonatal condition which would adversely affect the nutritional status of the newborn, (2) maternal or neonatal congenital metabolic disease, which may affect enzymatic activity or expression, including antioxidant enzymes (3) use of medications (other than insulin) which may affect carbohydrate metabolism, gastrointestinal function, or carbohydrate digestion.

### 2.3. Collection and Processing of Colostrum Samples

Mothers collected 5 mL of their colostrum between the 3rd and 5th day postpartum in the morning hours (8 a.m. to 11 a.m.). The needs and best interests of the newborns were always prioritized. All mothers were instructed to express colostrum immediately after feeding the baby, either into sterile plastic containers using clean electric breast pumps or by self-pumping after hand sanitization. The colostrum samples were immediately separated into aliquots, frozen, and stored at −80 °C until the measurements. The material was stored under these conditions until the analysis of the parameters described below, but for no longer than 6 months.

### 2.4. Measurements

Before the determination, the samples of human milk were gradually thawed in the refrigerator and then centrifuged for 5 min at 10,000× *g* at 4 °C. The defatted supernatant was extracted into new test tubes, which were used for the analysis.

#### 2.4.1. Analysis of Total Antioxidant Capacity (TAC)

TAC in human milk was measured using a commercial TAC Assay Kit (cat. No.: MAK187, Sigma-Aldrich, St. Louis, MO, USA). This kit allows for the measurement of total antioxidant concentration, including both small molecule and protein antioxidants. In the assay, Cu^2+^ ions are reduced to Cu^+^ by antioxidants present in the sample. The resulting Cu^+^ ions then form a complex with a colorimetric probe, producing a broad absorbance peak at 570 nm. The intensity of this absorbance is directly proportional to the total antioxidant capacity, expressed in Trolox equivalents.

#### 2.4.2. Analysis of Catalase Activity (CAT)

CAT activity in human milk was measured using a commercial Catalase Assay Kit (cat.no.: 707002, Cayman Chemical, Ann Arbor, MI, USA). The assay was performed according to the manufacturer’s instructions. The kit determines CAT activity by leveraging the enzyme’s peroxidative function. The assay relies on the reaction between CAT and methanol in the presence of an optimal amount of hydrogen peroxide. This reaction generates formaldehyde, which is then detected colorimetrically using Purpald as the chromogenic reagent. Purpald reacts specifically with aldehydes to form a bicyclic heterocyclic compound that turns purple upon oxidation, with absorbance measured at 540 nm.

#### 2.4.3. Analysis of Superoxide Dismutase (SOD)

The total SOD activity in human milk was determined by a commercial kit Superoxide Dismutase Assay Kit (cat. no.: 706002, Cayman Chemical, Ann Arbor, MI, USA). The assay was performed according to the manufacturer’s instructions. The kit uses a tetrazolium salt to detect superoxide radicals formed through the reaction of xanthine oxidase and hypoxanthine. One unit of SOD is defined as the enzyme quantity required to achieve 50% dismutation of superoxide radicals, determined by a change in absorbance at 450 nm. The kit is capable of measuring all three isoforms of SOD.

#### 2.4.4. Analysis of Glutathione Peroxidase (GPx)

GPx activity in human milk was measured using a commercial Glutathione Peroxidase Assay Kit (Cat. no: 702102, Cayman Chemical, Ann Arbor, MI, USA). The assay was performed according to the manufacturer’s instructions. The kit assesses GPx activity indirectly through a coupled enzymatic reaction involving glutathione reductase (GR). In this process, hydroperoxides are reduced by GPx, producing oxidized glutathione (GSSG), which is then converted back to its reduced form by GR in the presence of NADPH. The conversion of NADPH to NADP^+^ results in a decline in absorbance at 340 nm. When GPx activity is the rate-limiting step, the rate of absorbance decrease at 340 nm is directly proportional to the GPx activity in the sample.

#### 2.4.5. Analysis of Basic Composition of Colostrum

Macronutrient concentrations in colostrum samples were determined using the MIRIS Human Milk Analyzer^®^ (Miris AB, Uppsala, Sweden), a mid-infrared (MIR) transmission spectrometer specifically designed for human milk analysis. The instrument quantifies fat, crude protein, true protein, carbohydrates, dry matter, and energy content, following the manufacturer’s instructions. The analyzer requires a minimum sample volume of 2 mL and measures components based on the absorption of mid-infrared light at specific wavelengths, which correspond to functional groups present in each macronutrient. To ensure sample homogeneity and accurate readings, milk aliquots were first warmed to approximately 40 °C using an air bath. Homogenization was performed using the MIRIS Ultrasonic Processor, as per the manufacturer’s guidelines, to minimize variability due to sample heterogeneity.

### 2.5. Statistical Analysis

Statistical analysis was performed using the STATISTICA 13.3 (StatSoft, Inc., Tulsa, OK, USA), Microsoft Excel for Microsoft 365 (Microsoft, Redmond, WA, USA) and R version 3.6.2 (R Core Team, 2013. R Foundation for Statistical Computing, Vienna, Austria). A significance threshold was set at α = 0.05, with *p*-values below this considered statistically significant. Data presentations included mean with standard deviation (SD), median with interquartile range (IQR), or counts with corresponding percentages, depending on the data type. Cluster analysis, as detailed in a separate manuscript, was also performed [26]. Cluster analysis was conducted using the Marczewski-Steinhaus (M-S) taxonomic approach, followed by dendrogram construction [27]. The validity of the clustering method was verified using the Expectation-Maximization (E-M) algorithm [28]. In summary, the data-processing proceeded as follows. First, univariate regression via a generalized linear model was used to examine how selected maternal factors influence neonatal anthropometrics and body composition. From this, three predictors emerged as the strongest: membership in the study group, a maternal history of hypothyroidism, and total weight gain during pregnancy. Next, cluster analysis was performed using the Marczewski–Steinhaus (M-S) taxonomic method, accompanied by dendrogram construction. To validate this taxonomic approach, an Expectation-Maximization (E-M) algorithm was applied. The current manuscript focuses on a comparative analysis of colostrum composition, emphasizing the clusters identified in the prior study.

Although more common clustering techniques are better known and more widely used, we chose the Marczewski–Steinhaus (M-S) method because of several distinct advantages. First, M-S emphasizes the actual similarity between objects, which tends to yield clusters with stronger internal homogeneity of features and clearer separation between clusters. Second, it is more flexible in terms of data assumptions: it does not require that the data follow a specific distribution or assume linear relationships, enabling it to capture more complex underlying structures. Finally, the output tends to be easier to interpret: the clusters produced are relatively straightforward to understand and communicate.

To compare data across study groups and clusters, the Kruskal–Wallis test was employed, contingent upon data distribution characteristics. The Shapiro–Wilk test assessed normality, while Levene’s test evaluated homogeneity of variances. Post hoc Dunn’s test was conducted, if the results of a Kruskal–Wallis test were statistically significant, to determine exactly which groups/clusters were different at level of α.

### 2.6. Data Collection

Data on mothers and their infants—including the course of pregnancy, antenatal history, labor, and the puerperium—were collected at the time of enrollment using a personal questionnaire and were supplemented by information from maternal medical records. All scientific information collected as part of this study was treated confidentially. A unique code was assigned to each study participant to ensure anonymity, and data were stored safely at the study site.

## 3. Results

### 3.1. General Characteristics of the Study Population

The general characteristics of the study population are shown in Table 1. The numbers of participants in the particular groups were as follows: *n* = 15 in the GDM G1 group, *n* = 41 in the GDM G2 group and *n* = 21 in the non-GDM group. The groups did not differ significantly in maternal age or child birth weight. However, pre-gestational BMI was significantly higher in the GDM G2 group compared to controls and postpartum BMI also differed between groups, with higher values observed in both GDM groups. Gestational age at delivery was significantly lower in the GDM G2 group compared to the control group. No significant differences were observed between groups in the distribution of hypothyroidism, mode of delivery, newborn gender or feeding method.

### 3.2. Analysis of Antioxidant Status and Basic Composition of Colostrum

The analysis of selected antioxidants in human milk revealed a significant difference in total antioxidant capacity (TAC) between groups (*p* = 0.001). Median TAC values were lowest in the GDM G2 group (3.7 [2.4] nmol/μL), compared to GDM G1 (4.6 [6.8] nmol/μL) and the control group, which had the highest TAC levels (5.2 [1.4] nmol/μL). No statistically significant differences were found between groups for the enzymatic antioxidants such as catalase activity, superoxide dismutase activity and glutathione peroxidase activity (Table 2).

Despite the calculated activities of the CAT, SOD and GPx enzymes appearing to be elevated in the GDM G1 group in comparison to the other groups, the multiple comparison test did not reveal any statistically significant differences between the values.

**Table 3:** Results of Dunn’s post hoc test (with Bonferroni correction) comparing total antioxidant capacity (TAC) in human milk across study groups: Control, GDM G1, and GDM G2.

Comparison	Z Value	Unadjusted *p*	Adjusted *p*
Control vs. GDM G1	1.61	0.107	0.320
Control vs. GDM G2	3.84	0.000	**0.000**
GDM G1 vs. GDM G2	1.61	0.107	0.321

Z value—standardized test statistic; *p*.unadj—unadjusted *p*-value; *p*.adj—*p*-value adjusted for multiple comparisons using Bonferroni correction. Statistically significant comparisons (*p* < 0.05) are shown in bold.

In the analysis of macronutrient composition of colostrum, significant differences were found between groups in terms of energy content (*p* = 0.048) and dry matter concentration (*p* = 0.015). The highest energy and dry matter values were observed in the control group. No statistically significant differences were found for total protein (*p* = 0.153), true protein (*p* = 0.141), fat (*p* = 0.673), or carbohydrate content (*p* = 0.650) (Table 4).

**Table 4:** Comparison of Colostrum Macronutrient and Energy Content Between Study Groups.

Components of HM	All Participants *n* = 77	GDM G1 *n* = 15	GDM G2 *n* = 41	Control Group *n* = 21	*p* Value
Total protein [g/dL]	2.1 (0.7)	2.2 (0.9)	2.0 (0.6)	2.3 (0.7)	0.153 ^b^
True protein [g/dL]	1.7 (0.6)	1.8 (0.8)	1.6 (0.5)	1.9 (0.9)	0.141 ^b^
Fat [g/dL]	1.9 (1.2)	1.7 (1.7)	1.8 (1.5)	2.1 (1.1)	0.673 ^b^
Energy [kcal/dL]	58.0 (13.0)	55.5 (14.0)	56.0 (13.0)	61 (11.5)	0.048 **^b^**
Carbohydrates [g/dL]	7.5 (0.5)	7.3 (0.5)	7.5 (0.6)	7.5 (0.3)	0.650 ^b^
Dry matter [g/dL]	12.0 (1.4)	11.7 (1.3)	11.8 (1.7)	12.9 (1.4)	0.015 ^b^

Values are presented as median (IQR). Statistical significance was set at *p* < 0.05. b—One-way ANOVA was used for comparison between groups. Dunn’s test revealed statistically significant differences in dry matter content of colostrum between the control group and both GDM G1 (*p* = 0.035) and GDM G2 (*p* = 0.034). No significant difference was observed between GDM subgroups. Regarding energy content (kcal), no statistically significant differences were found after correction. However, there was a trend toward significance in the comparison between control and GDM G1 (*p* = 0.056) (Table 5).

**Table 5:** Dunn’s Post Hoc Comparisons of Colostrum Composition Between Study Groups.

Variable	Comparison	Z Value	Unadjusted *p*	Adjusted *p*
Dry Matter	Control vs. GDM G1	2.524	0.012	0.035
	Control vs. GDM G2	2.530	0.011	0.034
	GDM G1 vs. GDM G2	−0.577	0.564	1.000
Energy	Control vs. GDM G1	2.355	0.019	0.056
	Control vs. GDM G2	1.845	0.065	0.195
	GDM G1 vs. GDM G2	−0.998	0.318	0.955

### 3.3. Cluster Analysis

In the referenced study, researchers identified three distinct patient clusters by analyzing maternal health factors, including gestational diabetes mellitus (GDM) status and thyroid function. The clustering methodology employed was previously detailed in an earlier publication [20].

Identified clusters were as follows:

Cluster 1: Mothers diagnosed with GDM who exhibited normal thyroid function (*n* = 41)

Cluster 2: Mothers with concomitant diagnoses of GDM and hypothyroidism (*n* = 13)

Cluster 3: Mothers without GDM or thyroid dysfunction, representing the healthy control group (*n* = 21)

Cluster 4: Mothers without GDM but with a history of hypothyroidism.

In the current study, there were *n* = 43 participants in ‘Cluster 1”, *n* = 13 participants in ‘Cluster 2’ and *n* = 21 participants in ‘Cluster 3’. No participants were classified as belonging to Cluster 4.

#### Antioxidant Status and Basic Composition of Colostrum According to Cluster Analysis

No statistically significant difference was found among clusters according to CAT, SOD and GPx activity. In contrast, TAC differed significantly across the study clusters (*p* = 0.001). The highest TAC was observed in Cluster 3 (median 5.2 nmol/μL), while the lowest was in Cluster 2 (median 3.6 nmol/μL) (Table 6).

**Table 6:** Cluster-Based Differences in Antioxidant Activity in Colostrum Samples.

Variable	All Participants *n* = 77	Cluster 1 *n* = 43	Cluster 2 *n* = 13	Cluster 3 *n* = 21	*p* Value
CAT activity [nmol/min/mL]	13.9 (29.3)	12.9 (30.2)	16.3 (26.3)	16.9 (27.2)	0.676 **^b^**
SOD activity [U/mL]	0.6 (0.4)	0.6 (0.5)	0.8 (0.4)	0.5 (0.3)	0.073 **^b^**
GPx activity [nmol/min/mL],	10.7 (8.9)	12.7 (9.2)	9.2 (7.6)	9.2 (7.9)	0.240 **^b^**
TAC [nmol/uL] = [mM]	4.6 (2.7)	4.1 (3.0)	3.6 (1.9)	5.2 (1.4)	**0.001** ^b^

Values are presented as median (IQR). Statistical significance was set at *p* < 0.05. b—One-way ANOVA was used for comparison between groups. Dunn’s test showed statistically significant differences in TAC between cluster 1 and cluster 3 (*p* = 0.006), as well as between cluster 2 and cluster 3 (*p* = 0.003). No significant difference was observed between cluster 1 and cluster 2 (*p* = 0.819) (Table 7).

**Table 7:** Dunn’s Post Hoc Comparisons of Colostrum Composition Between Study Clusters.

Comparison	Z Value	Unadjusted *p*	Adjusted *p*
Cluster 1 vs. 2	1.07	0.273	0.819
Cluster 1 vs. 3	−3.083	0.002	0.006
Cluster 2 vs. 3	−3.309	0.001	0.003

The analysis of colostrum composition across clusters demonstrated significant differences in energy content (*p* = 0.047) and dry matter concentration (*p* = 0.011), while no significant differences were observed in the other macronutrients (Table 8).

**Table 8:** Comparison of Colostrum Macronutrient and Energy Content Between Clusters.

Components of HM	All Participants *n* = 77	Cluster 1 *n* = 43	Cluster 2 *n* = 13	Cluster 3 *n* = 21	*p* Value
Total protein [g/dL]	2.1 (0.7)	2.1 (0.6)	2.1 (0.7)	2.3 (0.7)	0.258 ^b^
True protein [g/dL]	1.7 (0.6)	1.7 (0.5)	1.7 (0.7)	1.9 (0.9)	0.219 ^b^
Fat [g/dL]	1.9 (1.2)	1.8 (1.4)	1.5 (1.1)	2.1 (1.1)	0.173 ^b^
Energy [kcal/dL]	58.0 (13.0)	56.0 (13.5)	55.5 (15.5)	61.0 (11.5)	**0.047 ^b^**
Carbohydrates [g/dL]	7.5 (0.5)	7.5 (0.6)	7.3 (0.4)	7.5 (0.3)	0.488 ^b^
Dry matter [g/dL]	12.0 (1.4)	11.8 (1.6)	11.5 (1.9)	12.9 (2.2)	**0.011 ^b^**

Values are presented as median (IQR). Statistical significance was set at *p* < 0.05. b—One-way ANOVA was used for comparison between groups. Dunn’s test revealed significant differences in dry matter content of colostrum between clusters. Specifically, dry matter levels were significantly higher in cluster 3 compared to both cluster 1 (*p* = 0.043) and cluster 2 (*p* = 0.018). No statistically significant difference was found between clusters 1 and 2. For energy content (kcal), no statistically significant differences were observed after adjustment, although a trend toward significance was noted between cluster 2 and cluster 3 (*p* = 0.058) (Table 9).

**Table 9:** Dunn’s Post Hoc Comparisons of Colostrum Composition Between Clusters.

Variable	Comparison	Z Value	Unadjusted *p*	Adjusted *p*
Dry Matter	Cluster 1 vs. 2	1.007	0.314	0.942
	Cluster 1 vs. 3	−2.448	0.014	**0.043**
	Cluster 2 vs. 3	2.750	0.006	**0.018**
Energy	Cluster 1 vs. 2	1.025	0.305	0.916
	Cluster 1 vs. 3	−1.880	0.060	0.180
	Cluster 2 vs. 3	−2.338	0.019	0.058

## 4. Discussion

The primary goal of GDM management is to maintain blood glucose levels within normal limits in order to reduce the risk of complications for both mother and infant. While many women achieve glycemic control through dietary modifications and physical activity, those who do not respond adequately within approximately two weeks typically require insulin therapy in addition to lifestyle changes [29,30]. By comparing these two subgroups, we aimed to explore whether the severity of glucose intolerance and the type of treatment received could impact the antioxidant and nutritional properties of colostrum.

The results revealed a significant difference in the total antioxidant capacity (TAC) of colostrum among the studied groups. The lowest TAC levels were observed in mothers with insulin-treated gestational diabetes (GDM G2), followed by the diet-controlled GDM group (GDM G1), with the highest levels found in the control group. Therefore, we hypothesize that the relatively higher antioxidant capacity observed in the diet-controlled GDM group (GDM G1) reflects the influence of healthier dietary habits and more balanced nutritional intake. In contrast, the lowest TAC levels found in the insulin-treated group (GDM G2) may suggest that more severe forms of GDM are associated with a greater degree of metabolic dysregulation, greater oxidative imbalance and reduced antioxidant defense mechanisms.

Our findings are partially consistent with those reported by Churchill et al. [24], who assessed oxidative stress markers in plasma and breast milk among women with gestational diabetes mellitus (GDM), distinguishing between diet-controlled and insulin-treated groups. The study involved 8 mothers who provided both breast milk and plasma samples, 34 mothers who donated only plasma samples, and 10 normoglycemic mothers who donated breast milk samples. In contrast to our results, authors found no statistically significant differences in the oxygen radical absorbance capacity (ORAC) values of breast milk between the study groups. While ORAC and total antioxidant capacity (TAC) are both used to assess antioxidant potential, they differ in scope: ORAC specifically measures the ability to neutralize peroxyl radicals—key contributors to lipid peroxidation—whereas TAC includes a broader spectrum of assays that evaluate the overall capacity to scavenge various free radicals. Nonetheless, consistent with our findings, Churchill et al. reported no significant differences in the activities of individual enzymatic antioxidants in breast milk across GDM subgroups. However, it is worth noting that in our study, although the differences did not reach statistical significance, higher concentrations of SOD, CAT, and GPx were observed in the GDM G1 group compared to the GDM G2 group.

In another study by Zygula et al. [31] where oxidative stress markers in plasma between women with and without GDM were measured, women with GDM G1 exhibited significantly higher plasma ORAC compared to both the GDM G2 group and normoglycemic controls. The authors hypothesized that the higher antioxidant status observed in the diet-controlled group might result from more balanced dietary practices, potentially motivated by the desire to avoid glycemic instability. Interestingly, our results in colostrum reflect a similarly elevated antioxidant capacity in the GDM G1 group suggesting that the improved systemic antioxidant status in the diet-controlled group may be reflected in breast milk composition as well. This alignment supports the hypothesis that maternal metabolic regulation and dietary behavior can influence the antioxidant profile of colostrum.

Our results show that the energy and dry matter content of colostrum were significantly lower in the groups of women with gestational diabetes (GDM), compared to the control group. Interestingly, no significant differences were observed in the major macronutrients, such as protein, fat, or carbohydrates. This suggests that while the qualitative composition of breast milk remains relatively stable, quantitative variations in energy density and dry matter may occur depending on maternal metabolic status. These findings partially contrast with those reported in the most recent systematic review and meta-analysis by Qin et al. [32], who found that, compared to non-GDM women, the colostrum of women with GDM had a significantly higher protein content (*p* = 0.03), with no significant differences in carbohydrate, lipid, or energy levels. Simultaneously, the mature milk of GDM women had a higher protein content (*p* = 0.007) and a higher lipid content (*p* = 0.001), with no significant differences in carbohydrates and energy. This underscores the need for further research into the impact of maternal metabolic disorders on milk composition and their potential consequences for infant development.

Despite the lack of statistically significant differences in the activities of individual antioxidant enzymes: CAT, SOD and GPx—across the identified clusters, our findings revealed a significant variation in TAC, suggesting broader differences in the antioxidant profile of colostrum. Notably, Cluster 3, comprising healthy mothers without gestational diabetes mellitus or thyroid dysfunction, exhibited the highest TAC, even though their enzymatic antioxidant activities were relatively low or moderate. This observation points to a potentially greater contribution of non-enzymatic antioxidants—such as vitamins C and E, lactoferrin, polyphenols, etc.—in enhancing the overall antioxidant defense in this group. These components are not captured by enzymatic assays, highlighting the importance of evaluating both enzymatic and non-enzymatic factors when assessing antioxidant capacity. The elevated TAC in Cluster 3 may be influenced by favorable maternal or environmental conditions, including a balanced diet, lower oxidative stress, healthier gut microbiota, or better-controlled inflammatory status during the perinatal period. These factors may promote the synthesis and secretion of non-enzymatic antioxidants into colostrum, thus strengthening neonatal protection during the early postnatal phase. In contrast, Cluster 2, which included mothers with both GDM and hypothyroidism, demonstrated the lowest TAC. Interestingly, this group also showed slightly elevated SOD activity, which might represent a compensatory response to increased oxidative stress. It remains unclear whether the upregulation of enzymatic defense mechanisms in this cluster is sufficient to counterbalance the reduced total antioxidant potential, or if these women are at risk of producing colostrum with suboptimal protective properties. Cluster 1, composed of mothers diagnosed with GDM but with normal thyroid function (*n* = 41), presented intermediate TAC values and enzyme activity levels, further underscoring the potential impact of thyroid dysfunction on antioxidant status in lactating individuals.

Results regarding macronutrients content revealed that energy and dry matter content in colostrum varied significantly between study clusters, while levels of protein, fat and carbohydrates remained relatively consistent. The higher energy and dry matter content observed in Cluster 3, may reflect more favorable maternal metabolic conditions or optimal mammary gland function in healthy mothers without GDM or thyroid dysfunction and could suggest potentially better early postnatal nutrition for the infant.

There is a growing body of evidence to suggest a link between GDM and elevated oxidative stress, along with modifications to antioxidant defence mechanisms. Research has demonstrated that women diagnosed with GDM exhibit diminished activities of SOD and GPx, concomitant with augmented CAT activity, suggesting an imbalance in redox homeostasis. This imbalance has the potential to exacerbate lipid peroxidation and contribute to complications during pregnancy. In addition, an investigation into the antioxidant capacity of breast milk in women diagnosed with gestational diabetes mellitus (GDM) reveals a significant reduction in antioxidant levels. This decline has the potential to impact the oxidative stress status of the newborn, necessitating further research in this area [33,34]. Elevated thyroid-stimulating hormone (TSH) levels have been demonstrated to be associated with increased oxidative stress and decreased activities of antioxidant enzymes such as SOD, CAT, and GPx. This oxidative imbalance may have implications for both maternal health and the antioxidant quality of breast milk. Monitoring the levels of SOD, CAT, GPx, and TAC in breast milk can provide insights into the oxidative status of mothers with GDM and thyroid disorders. Addressing oxidative stress through nutritional interventions or antioxidant supplementation may improve maternal health outcomes and enhance the antioxidant properties of breast milk, thereby supporting neonatal development and reducing the risk of oxidative stress-related complications in infants [35,36]. It is evident that the antioxidant and anti-inflammatory components present in breast milk have a significant impact on epigenetic regulation and immune system programming. This, in turn, establishes the foundation for disease resistance and metabolic health, which is why they play a crucial role in nutritional and metabolic programming [37].

Our study has several limitations that should be considered when interpreting the results. Complexity of human milk biological matrix poses significant analytical challenges when measuring biochemical parameters such as total antioxidant capacity. Therefore, careful sample preparation and rigorous validation of analytical methods are essential to ensure reliable measurements. In our study, no additional validation of the assay kit used was performed. However, the kit is designed for a wide range of biological matrices, and defatting of samples substantially reduced the potential matrix effects.

One of the most important limitations of this study is the absence of maternal dietary assessment. While the antioxidant potential of colostrum was analyzed biochemically, no dietary intake data—such as food frequency questionnaires or dietary recall—were collected. Given that maternal diet can significantly influence the antioxidant profile of breast milk, the lack of this information limits the ability to fully interpret the nutritional and metabolic context of the findings. Additionally, the study did not assess the use of dietary supplements, such as vitamins, minerals, or antioxidant preparations, which could also affect the antioxidant capacity of colostrum. This may represent a potential confounding factor. Future research should include standardized dietary assessment tools and data on supplement use to provide a more comprehensive understanding of the maternal factors influencing breast milk composition.

Another important limitation is the small sample size. Although the results give an overview of the problem addressed, there is a need to verify these results on a larger number of participants. While this study focused on measuring total antioxidant capacity of colostrum, it did not assess specific markers of oxidative stress. Future research should consider including established oxidative stress biomarkers, such as malondialdehyde (MDA), 8-hydroxydeoxyguanosine (8-OHdG), advanced oxidation protein products (AOPP), and protein carbonyls, to provide a more comprehensive understanding of redox balance in women with gestational diabetes mellitus [38]. Additionally, colostrum samples were collected at a single time point postpartum, which may not capture dynamic changes in milk composition over time. In addition, it is not possible to guarantee the replicability of the results. There is also a risk that assumptions made on the basis of the data obtained are inaccurate. Another limitation of this study is the lack of comparable research in this area. So far, only one study has investigated the relationship between gestational diabetes mellitus (GDM), antioxidant status, and breast milk composition. Consequently, it was not possible to directly compare the present findings with a broader body of literature. This significantly limits the contextual interpretation of the results and underlines the need for further research to validate and expand upon these preliminary observations.

## 5. Conclusions

This study provides new insights into the antioxidant profile of colostrum in the context of GDM. Our findings suggest that maternal metabolic and endocrine conditions, such as GDM and thyroid dysfunction, may differentially influence the nutritional and functional properties of colostrum—particularly its antioxidant potential. Severe GDM (G2) has been demonstrated to be associated with diminished total antioxidant capacity and reduced energy/dry matter in colostrum. This has the potential to diminish oxidative protection and energy availability for newborns, particularly during the delicate colostrum phase, which is characterized by its high antioxidant and bioactive content. The presence of unaltered enzymatic antioxidants and macronutrient ratios indicates that the observed deficiencies are not indicative of a global loss of milk quality but rather point to specific deficits in energy density and TAC.

## Figures and Tables

**Table 1:** Characteristics of study participants and their neonates.

Feature	All Participants *n* = 77	GDM G1 *n* = 15	GDM G2 *n* = 41	Control Group *n* = 21	*p*-Value
Age (years), Mean (SD)	33.5 (4.8)	32.5 (5.7)	34.1 (4.0)	32.8 (5.6)	0.622 ^b^
Maternal pre-gestational BMI, Median (IQR)	24.4 (4.5)	24.6 (4.7)	26.2 (5.5)	23.1 (3.6)	0.003 ^a^
Maternal post-partum BMI, Median (IQR)	28.0 (6.1)	28.4 (9.0)	28.3 (4.5)	26.9 (5.3)	0.028 ^a^
Gestational weight gain:					
within normal limits	59 (76.6)	9 (11.7)	32 (41.6)	18 (23.4)	
excessive	18 (23.4)	6 (7.8)	9 (11.7)	3 (3.9)	0.189 ^c^
Maternal hypothyroidism, n (%)	13 (16.9)	2 (2.6)	11 (14.3)	0 (0.0)	0.261 ^c^
Gestational age at birth (weeks), Median (IQR)	39 (1.0)	39 (2.0)	38 (1.0)	40 (2.0)	0.0002 ^a^
Mode of delivery:					
vaginal	35 (45.5)	6 (7.8)	20 (26.0)	9 (11.7)	
cesarean section	42 (54.5)	9 (11.7)	21 (27.2)	12 (15.6)	0.811 ^c^
Assigned child gender:					
female	41 (53.2)	10 (12.9)	20 (26.0)	11 (14.3)	
male	36 (46.8)	5 (6.5)	21 (27.3)	10 (13.0)	0.492 ^c^
Child birth weight (kg), Mean (SD)	3.4 (0.5)	3.4 (0.5)	3.3 (0.6)	3.4 (0.4)	0.800 ^b^
Method of feeding of a newborn, n (%):					
Exclusive breastfeeding	45 (58.4)	9 (11.7)	27 (35.0)	9 (11.7)	0.218 ^c^
Mixed breastfeeding	32 (41.6)	6 (7.8)	14 (18.2)	12 (15.6)	

Data are presented as *n* (%) or mean ± SD (median, IQR). The *p*-value < 0.05 was considered statistically significant. a—Kruskal–Wallis test; b—one-way ANOVA; c—Chi-square test.

**Table 2:** Antioxidants activity and total antioxidant capacity (TAC) of colostrum samples according to study groups.

Variable	All Participants *n* = 77	GDM G1 *n* = 15	GDM G2 *n* = 41	Control Group *n* = 21	*p* Value
CAT activity [nmol/min/mL]	13.9 (29.3)	20.3 (24.0)	9.7 (24.5)	16.9 (27.2)	0.349
SOD activity [U/mL]	0.6 (0.4)	0.9 (0.5)	0.6 (0.4)	0.5 (0.3)	0.052
GPx activity [nmol/min/mL]	10.7 (8.9)	14.5 (7.4)	9.7 (9.4)	9.2 (7.9)	0.213
TAC [nmol/uL] = [mM]	4.6 (2.7)	4.6 (6.8)	3.7 (2.4)	5.2 (1.4)	0.001

Data are presented as median, IQR. The *p*-value < 0.05 was considered statistically significant. Dunn’s post hoc test with Bonferroni correction was used to evaluate potential differences in colostrum composition across groups. Dunn’s test revealed a statistically significant difference in TAC between the control group and the GDM G2 group (adjusted *p* = 0.000). No statistically significant differences were observed between the control group and GDM G1 (adjusted *p* = 0.320) or between GDM G1 and GDM G2 (adjusted *p* = 0.321) (Table 3).

## Data Availability

The data presented in this study are available upon reasonable request from the corresponding author.

## Abbreviations

The following abbreviations are used in this manuscript:

AAP	American Academy of Pediatrics
CAT	catalase
CVD	cardiovascular disease
ESPGHAN	European Society for Paediatric Gastroenterology, Hepatology and Nutrition
GDM	gestational diabetes mellitus
GPx	glutathione peroxidase
LGA	large for gestational age
RDS	respiratory distress syndrome
ROS	reactive oxygen species
SOD	superoxide dismutase
TAC	Total Antioxidant Capacity
UTIs	urinary tract infections
WHO	World Health Organization
